# Unraveling plankton adaptation in global oceans through the untargeted analysis of lipidomes

**DOI:** 10.1126/sciadv.ads4605

**Published:** 2025-05-23

**Authors:** Weimin Liu, Henry C. Holm, Julius S. Lipp, Helen F. Fredricks, Benjamin A. S. Van Mooy, Kai-Uwe Hinrichs

**Affiliations:** ^1^ Organic Geochemistry Group, MARUM—Center for Marine Environmental Sciences, University of Bremen, Bremen 28359, Germany.; ^2^ Department of Marine Chemistry and Geochemistry, Woods Hole Oceanographic Institution (WHOI), Woods Hole, MA 02543, USA.; ^3^ Lamont-Doherty Earth Observatory, Columbia University, Palisades, NY 10964, USA.

## Abstract

Microbial responses to environmental changes are well studied in laboratory cultures, but in situ adaptations of plankton lipidomes remain less understood. Building upon a global lipidomic study showing temperature-driven lipid unsaturation regulation in marine plankton, we expanded the analysis spatially and methodologically to investigate the in situ adaptations of marine plankton. Through weighted correlation network analysis of 3164 lipid species from 930 samples, we identified 16 structurally distinct lipid clusters co-occurred across diverse oceanographic conditions. The highest lipid diversity was observed in the polar oceans, where plankton uses chain shortening for cold acclimation. Conversely, in the surface of tropical and subtropical oceans, plankton showed enrichment in non-phosphorus lipids, likely responding to warm temperature, with potential implications for the elemental stoichiometry of the biological pump. In the subsurface of these regions, highly unsaturated lipids were enriched, suggesting phytoplankton adaptation to low light and contributing unsaturated fatty acids to tropical and subtropical ocean food webs.

## Teaser

Lipidomic study shows plankton adaptations to temperature, light, and nutrient changes on a global scale.

## INTRODUCTION

Lipids are essential components of cellular membranes in marine organisms, playing a crucial role in maintaining membrane fluidity, mediating nutrient uptake, and adapting to environmental changes (*1*). Lipids, particularly intact polar lipids that consist of lipid head groups and fatty acids tails, serve as valuable biomarkers for characterizing and identifying microbial groups in marine ecosystems, enabling the reconstruction of microbial community structure and function (*2*, *3*). Understanding the distribution and dynamics of lipids in marine environments is crucial as it allows us to unravel ecological dynamics and biogeochemical processes occurring in global oceans. This knowledge is essential for predicting how marine ecosystems respond to environmental changes and for understanding the fundamental processes that regulate global biogeochemical cycles (*4*–*6*).

The upper ocean is rich in diverse classes of lipids, associated with various biological functions. Because of their vital role as the interface between cells and their surrounding environments, lipids have received notable attention as indicators of microbial community dynamics in studies of the marine water column and sediments. However, lipids often resist simple classification into taxonomically defined sources or environmentally driven adaptations (*7*). Certain lipids carry taxonomic information: For example, ornithine-containing lipids are widespread among bacteria but are absent in Archaea and Eukarya (*8*). Nonetheless, studies have revealed that the polar head groups and the core structures of diverse glycerolipids are subject to modifications, enabling organisms to better adapt to changing environmental conditions (*9*). For instance, living organisms can modulate the degree of unsaturation of acyl groups in response to temperature changes, a phenomenon known as homeoviscous adaptation (*10*). Such adaptation was recently observed on a global scale in a study by Holm *et al.* (*11*), which investigated the average unsaturation of lipid species from ten glycerolipid classes across global oceans, showing that fatty acid unsaturation is fundamentally linked to ocean temperature.

The interplay between environmental factors and the collective lipidomic response could result either from the regulation of genes caused by environmental forcing or from the selective survival of organisms that constitutively have lipid profiles suited to thrive in specific environmental conditions (*12*, *13*). Weighted correlation network analysis (WGCNA) has been extensively used in genomics to group functionally related genes into modules and establish connections with traits and environmental factors (*14*). WGCNA can be extended to lipidomics to group lipids, exploring microbial origins and their responses to environmental changes (*15*). Unlike networks based on molecular structure similarity—such as feature-based molecular networking (*16*, *17*), a common untargeted approach in environmental lipidomics (*18*, *19*)—WGCNA identifies connections between lipids by examining their similarity in spatial distribution patterns within specific environmental contexts.

In this study, we apply WGCNA to the extensive dataset published by Holm and Van Mooy (*20*). This dataset comprises samples collected from seven research cruises across the Atlantic, North Pacific, and the Antarctic Oceans, providing lipidome profiles from a variety of oceanic zones including mesopelagic regions extending from the surface to 200-m depth and parts of the epipelagic regions reaching up to 400-m depth. In addition, it spans a range of latitudes from polar to tropical oceans and includes both nutrient-rich and nutrient-depleted environments. By leveraging this dataset, we aim to investigate the intricate relationships between lipids and environmental factors on a global scale. Specifically, we seek to elucidate how marine plankton adapt to environmental changes, with a particular focus on the influence of temperature variations, nutrient availability, and light availability.

## RESULTS

### Eigenlipids and their spatial distribution

**Fig. 1.**
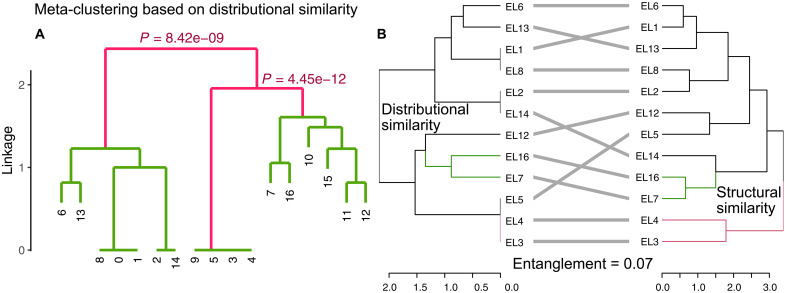
Meta-clustering of EL clusters based on distributional similarity and their entanglement with structural similarity. (**A**) Meta-clustering of the EL clusters based on distributional similarity, integrating the WGCNA clustering results, distribution across Longhurst provinces and depth profiles. Red lines indicate statistically significant nodes, with corresponding *P* values shown. Digits represent EL clusters. (**B**) Tanglegram comparing hierarchical clustering dendrograms derived from distributional similarity (left) and structural similarity (right). The dendrogram for distributional similarity has a cophenetic correlation coefficient of 0.90, while the structural similarity dendrogram has a cophenetic correlation coefficient of 0.82. The entanglement score between the two dendrograms is 0.07. Colored branches indicate consistent clustering patterns observed in both dendrograms.

A total of 3164 lipid species were included in this study. These include 847 lipid species identified in Holm *et al.* (*11*), designated as annotation type 1 (Ann.1; see Materials and Methods), with the lipid classes listed in table S1. In addition, 1486 lipid species were identified using LOBSTAHS (*21*) and LIPIDMAPS (*22*), designated as Ann.2. The remaining 831 lipid species, which do not belong to Ann.1 or Ann.2 but yielded MS^2^ spectra, are designated as Ann.3. In this study, “eigenlipids” (ELs) refer to clusters of lipids with similar distribution patterns, characterized by correlated peak intensities across samples. The analysis of these lipid species yielded 16 reproducible (text S1) co-occurring ELs, which are ordered by the number of included lipid species. In addition, a collection of lipid species, labeled as EL0, comprised species that were not associated with any cluster (*14*). To simplify interpretation, these ELs were further grouped into three metaclusters, as shown in Fig. 1A: meta-cluster 1 (MC1), comprising EL1, EL2, EL6, EL8, EL13, EL14, and EL0; meta-cluster 2 (MC2), including EL3 to EL5 and EL9; and meta-cluster 3 (MC3), consisting of EL7, EL10 to EL12, EL15, and EL16.

**Fig. 2.**
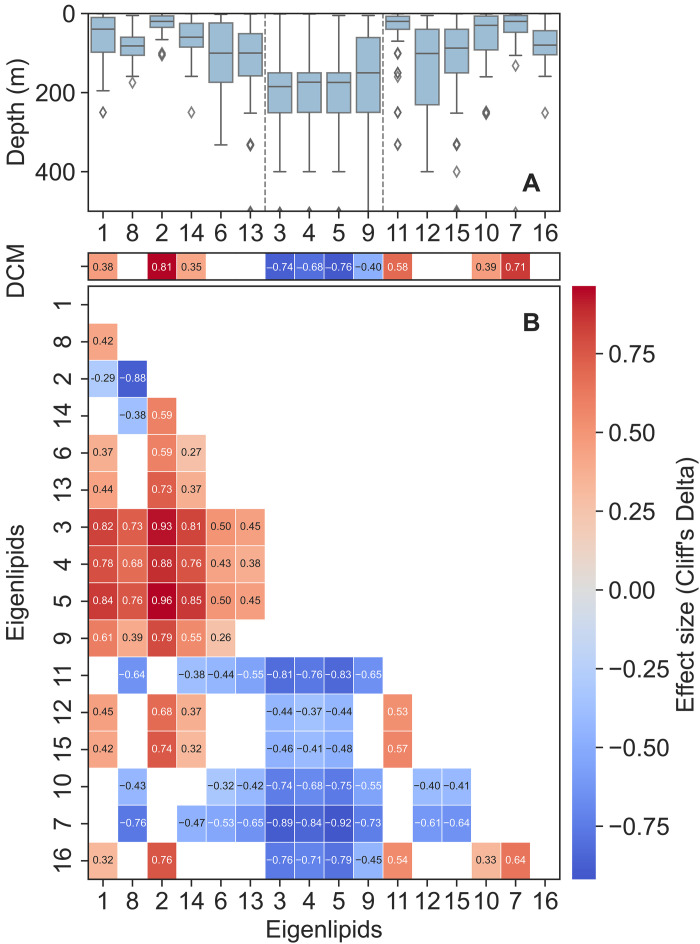
Vertical distribution patterns of ELs. (**A**) Depth distribution where each EL reaches its highest intensity in the water column (the “EL maximum depth”), highlighting depth specificity across the oceans. ELs on the *x* axis are ordered on the basis of meta-clustering similarities. In each boxplot, the central line represents the median, the box spans the interquartile range (IQR; 25th to 75th percentiles), and the whiskers extend to 1.5× the IQR. Data points outside this range are plotted as individual outliers. (**B**) Heatmap showing pairwise comparisons of EL maximum depths and comparisons between EL maximum depths and the chlorophyll maximum (DCM) depth. Only significant comparisons (adjusted *P* < 0.05) are displayed, with effect sizes (Cliff’s Delta) ranging from −1 to 1. Positive values indicate deeper depths for the EL or DCM on the *y* axis than for the EL on the *x* axis, while negative values indicate the opposite.

**Fig. 3.**
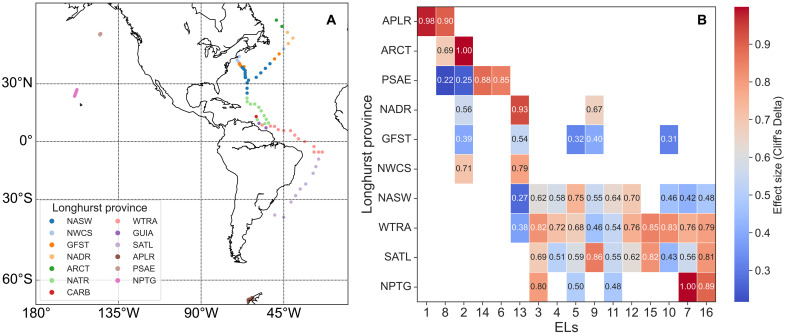
Prevalence of ELs in the mixed layer across Longhurst biogeochemical provinces. (**A**) Sampling locations, where each site is color-coded by its corresponding Longhurst biogeochemical province. (**B**) Heatmap showing the Longhurst provinces in which each EL is significantly more prevalent (adjusted *P* < 0.05) compared to all other provinces. The heatmap is color-coded by effect size (Cliff’s Delta), ranging from 0 to 1, with higher values indicating greater differences in EL intensity for the highlighted province relative to all others. APLR, Austral polar; ARCT, Atlantic Arctic; CARB, Caribbean; GFST, Gulf Stream; GUIA, Guianas coast; NADR, North Atlantic Drift; NASW, Northwest Atlantic subtropical gyre; NPTG, North Pacific tropical gyre; NATR, North Atlantic tropical gyre; NWCS, Northwest Atlantic shelves; WTRA, Western tropical Atlantic; PSAE, Eastern Pacific subarctic gyre; SATL, South Atlantic gyre.

The relative intensities of these ELs, calculated by dividing each EL’s total lipid peak intensity by the total peak intensity of all annotated lipid species, are shown in fig. S1. This provides a semiquantitative overview of the relative contribution of each EL to the overall lipidome. These ELs exhibit remarkable variability in both vertical and geographical distributions (fig. S2) across the global oceans. To quantify vertical variability, the depth at which each EL reaches its highest relative intensity (EL maximum depth) at each sampling station was calculated and compared (Fig. 2). Geographical variability was assessed by analyzing spatial patterns within the framework of Longhurst biogeochemical provinces (Fig. 3A) (*23*). Provinces where each EL is significantly more enriched than at other provinces are exhibited in Fig. 3B. In addition, the relationship between the relative intensities of ELs and environmental parameters were explored and visualized using principal components analysis (PCA), as shown in fig. S3, providing further insights into the links between EL distributions and environmental conditions.

### Structural dissimilarity of eigenlipids

In addition to their spatial patterns, ELs show variance in lipid composition (tables S2 and S3), including differences in headgroup composition (fig. S4), fatty acid profiles (fig. S5), and other molecular properties (fig. S6). Structural dissimilarity among ELs was quantified using mixed-effects modeling (fig. S7). Because of the limited number of lipid species with annotated headgroups, EL9 to E11 and EL15 were excluded from the structural dissimilarity analysis. The primary structural dissimilarity among ELs was in their fatty acid profiles, followed by variations in headgroup composition. The resulting structural dissimilarity clustering was compared with the distributional similarity–based clustering in Fig. 1B. This comparison, presented as a tanglegram, yielded an entanglement score of 0.07, indicating a strong agreement between the two clusterings (the entanglement score is between 0 and 1, with lower scores reflecting better alignment).

### Meta-cluster 1

Geographically, all MC1 clusters, except EL13, are most prevalent in the sampled polar or subpolar provinces (Fig. 3B). EL1 and EL8 both exhibit the most significant enrichment in the Austral Polar Province (APLR). EL1 includes the largest number of lipid species, suggesting numerically the highest lipid diversity in APLR. EL2 is most prevalent in the Atlantic Arctic, while both EL6 and EL14 are most prevalent in the Eastern Pacific Subarctic gyre. Vertically, the depths at which EL6, EL8, and EL13 reach their maximum peak intensities are significantly deeper than those of the other MC1 clusters and are not significantly different from the depth of deep chlorophyll maximum (DCM) (Fig. 2B). The DCM zone is identified either using conductivity-temperature-depth (CTD) data or inferred from the peak intensity of chlorophyll-*a* where CTD data are unavailable. Furthermore, the relative intensity of EL8 exhibits a positive correlation with chlorophyll-*a* fluorescence (fig. S8A).

Structurally, all MC1 cluster, except EL14, are characterized by the predominance of polyunsaturated fatty acids (PUFAs), despite variations in headgroup compositions. EL1 and EL6 cluster together early (Fig. 1B). EL1 contains significantly more triacylglycerols (TAGs), phospholipids including phosphatidylglycerol (PG) and phosphatidylcholine (PC), and glycolipids including sulfoquinovosyl diacylglycerol (SQDG) and digalactosyldiacylglycerol (DGDG). EL6 contains significantly more TAGs and PCs. EL1 and EL6 are subsequently joined by EL13, which contains phospholipids (table S2), although not significantly different from other ELs. EL2 is exclusively dominated by non-phosphorus lipids, including both betaine and glycolipids. EL8 contains primarily chloroplast lipids. These two clusters cluster together and join EL1, EL6, and EL13. EL14 is structurally distant from other ELs and characterized by relatively shorter chain length (fig. S5A).

### Meta-cluster 2

MC2 clusters are characterized by their significantly higher peak intensities at the bottom of epipelagic zone and at the top of mesopelagic zone. They all reach maximum peak intensity at depths significantly deeper than DCM (Fig. 2B).

Structurally, EL3 and EL4 are closely clustered, characterized by the absence of glycolipids (fig. S4), the predominance of saturated fatty acids (SFAs) and monounsaturated fatty acids (MUFAs) (fig. S5B), and a relatively large fraction of odd-numbered fatty acid chains (fig. S7D). Phospholipids are found in EL4 and EL5, with PG being the major phospholipid in EL4, and phosphatidylethanolamine (PE), PC, and PG being the major phospholipids in EL5. EL3 contains primarily betaine lipids, along with a large amount of highly saturated neutral lipids such as TAGs. EL9 contains few glycerolipids (table S2) and appears to be absent in phospholipids (fig. S6D).

### Meta-cluster 3

EL10 and EL11 appear to be region specific. EL10 exhibits a relatively higher intensity in Western Tropical Atlantic near the South American coast (fig. S2J). EL11 shows a relatively higher intensity in the Northwest Atlantic subtropical gyre and, more specifically, the Sargasso Sea (fig. S2K). Both EL12 and EL15 exhibits relatively higher intensity in the tropical Atlantic than the other regions, while EL7 and EL16 exhibits pronounced prevalence not only in tropical Atlantic but also in North Pacific Tropical gyre. The relative intensity of EL7 has the longest projection onto the loading vector of in situ temperature in the PCA biplot (fig. S3), indicating the strongest association between EL7 and in situ temperature. The depth at which EL7 reaches its maximum peak intensity is significantly shallower than the DCM, while that of EL16 is not significantly different from the DCM. In addition, the relative intensity of EL16 is not correlated with chlorophyll-*a* fluorescence but instead with a specific pigment, divinyl chlorophyll-*a* (fig. S8B). Furthermore, EL16 shows a strong positive correlation with the total biovolume of *Prochlorococcus* reported by Karlusich *et al.* (*24*) (fig. S9).

Structurally, EL7 and EL16 are closely related but distinct from EL12. EL7 is characterized by chloroplast lipids along with betaine lipids, while EL16 contains exclusively chloroplast lipids. In contrast, EL12 is dominated by TAGs (fig. S4). All three ELs share a predominance of SFA/MUFA chains; however, EL7 and EL16 have shorter carbon chains, with fewer than 16 carbon atoms per fatty acid on average (fig. S5A). None of the Ann.1 lipids are clustered within EL10 and EL15. In addition, the Ann.2 lipids from EL10 and EL11 are characterized by the absence of phosphorus in their elemental compositions (fig. S6D).

### Influence of temperature on the chain length of fatty acids

**Fig. 4.**
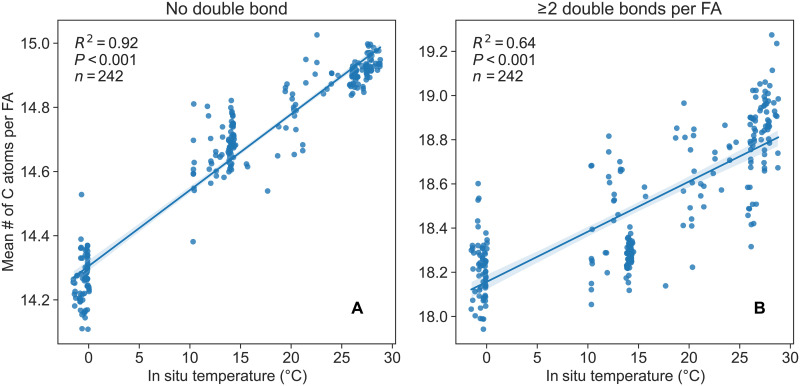
Mixed layer temperature is highly correlated with fatty acid chain length. Trends in fatty acid chain length as a function of in situ temperature across all membrane glycerolipids, including PC, PG, PE, SQDG, MGDG, DGDG, GADG (glucuronic acid diacylglycerol), DGTS/A (diacylglyceryl trimethylhomoserines and diacylglyceryl hydroxymethyltrimethyl-β-alanines), and DGCC (diacylglyceryl-3-O-carboxyhydroxymethylcholine), containing SFAs (**A**) and PUFAs (**B**) are shown. The weighted mean carbon number per fatty acid (FA) is plotted against in situ temperature for mixed layer samples. The blue line represents linear fits; the inset depicts the coefficient of determination for the linear regression fit using ordinary least squares.

A gradual increase in average carbon chain length is observed in ELs transitioning from those dominating polar provinces to those prevalent in subpolar provinces (fig. S5A). To determine whether this change in chain length is statistically significant, the relationship between temperature and the chain length of lipid species was examined, focusing on both SFAs and PUFAs. In both cases, strong positive correlations were observed (Fig. 4).

### Lipids enriched in tropical and subtropical oceans

**Fig. 5.**
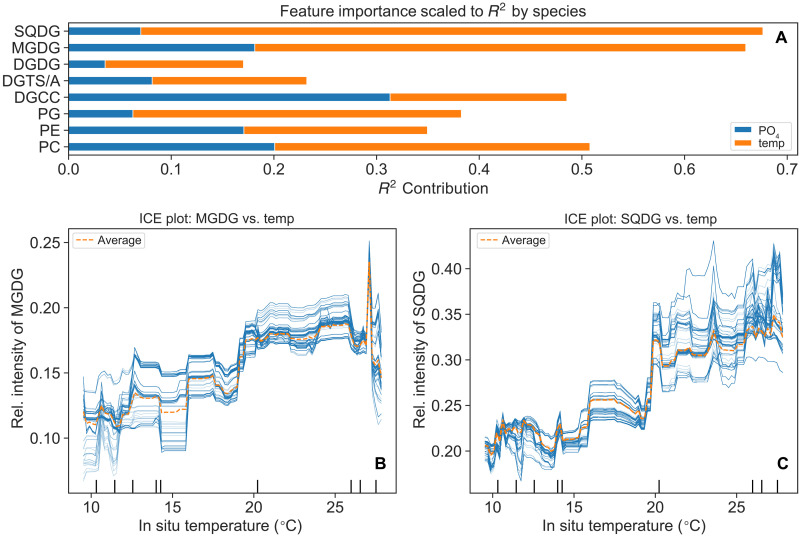
Increasing temperature drives the accumulation of glycolipids at the sea surface. Relative intensities were calculated by normalizing the intensities of each lipid class with SFA/MUFA chains to the total intensity of lipids containing SFA/MUFA chains. (**A**) *R*^2^ scores of the fitted Random Forest models for each lipid class, with bar plots indicating the contribution of PO_4_ concentration and in situ temperature to the model’s predictions. (**B**) and (**C**) Individual Conditional Expectation (ICE) plots showing the predicted relative intensities of the glycolipids MGDG and SQDG, respectively, as functions of in situ temperature, while holding PO_4_ concentration constant. Each curve in the ICE plot represents the response trajectory of an individual sample, highlighting the influence of in situ temperature on the accumulation of these lipids. ICE plots for other lipid classes are provided in fig. S11.

To evaluate the relative contributions of warm temperature and low nutrient availability to the enrichment of EL7 in the surface of tropical and subtropical ocean, a Random Forest regression model was applied. The result (fig. S10) indicates that changes in temperature explain most of the variance in the relative intensities of EL7 at the sea surface. To further investigate whether this enrichment is linked to specific headgroups besides the predominance of SFA/MUFA chains, Random Forest regression models were applied to different classes of lipids containing SFA/MUFA chains, including those not in EL7. The analysis indicated that, with the exception of MGDG 32:2, glycolipids MGDG and SQDG exhibit strong enrichment with increasing temperature (Fig. 5, B and C), while betaine lipids exhibit strong enrichment at extremely low phosphate concentrations (fig. S11, A and E).

### DCM-related eigenlipids

**Fig. 6.**
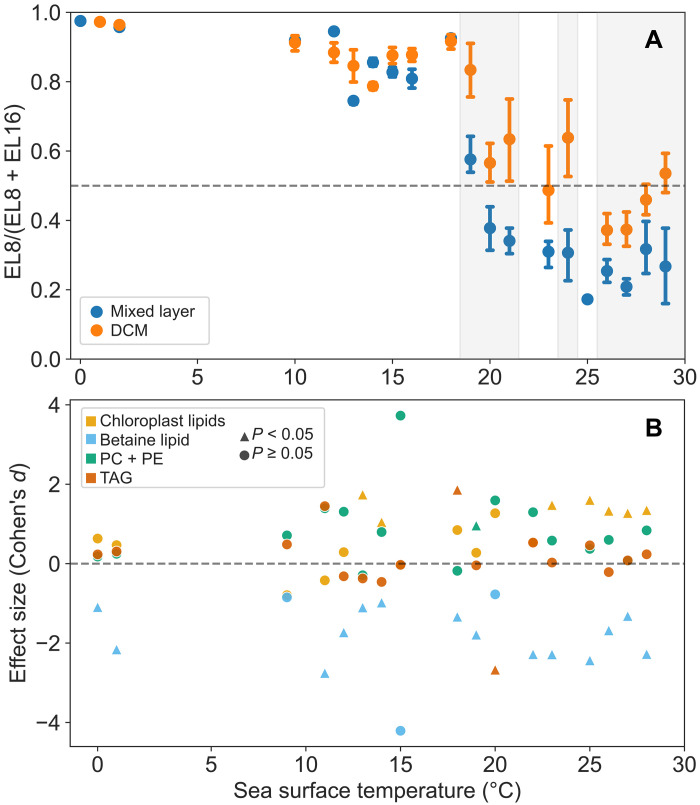
Enrichment of chloroplast-related PUFAs in the DCM of tropical and subtropical oceans. (**A**) Shifts in the predominance of two EL clusters dominated by chloroplast lipids, EL8 and EL16, from cold to warm sea surface temperatures (SST) across the global oceans in the mixed layer and the DCM. The ratio of the intensity of EL8 to the sum intensity of EL8 and EL16 is plotted on the *y* axis. Error bars show the 95% confidence interval of the estimated mean ratio at each SST. The gray-shaded area indicates a significantly higher ratio (adjusted *P* < 0.05) in the DCM than in the mixed layer. (**B**) Differences in PUFA-containing lipids between the mixed layer and the DCM for various lipid classes. The relative intensities for these classes were compared between the two layers. Triangular markers with positive effect sizes (Cohen’s *d*) indicate a significantly higher (adjusted *P* < 0.05) PUFAs in the DCM relative to the mixed layer. SQDG, MGDG, DGDG, and PG are included in the chloroplast lipids.

Among all ELs, EL6, EL8, EL12, EL13, EL15, and EL16 reach their maximum relative intensities at depths that are not significantly different from the DCM depth (Fig. 2B). Notably, EL8 and EL16 contain significantly more chloroplast lipids than all other DCM-related ELs. However, these two ELs show distinct fatty acid profiles (fig. S5, A and B), with EL8 containing primarily PUFAs. To further explore how these differences in fatty acid composition relate to their spatial distribution, the relative intensity of EL16 in the water column was compared to that of EL8 across global oceans (Fig. 6A). A significant increase in the ratio of EL8 to EL16 from mixed layer to DCM was observed in tropical and subtropical oceans. To determine whether this increase is specific to chloroplast lipids or reflects broader trends in PUFA distributions, the relative intensity of PUFAs categorized by different headgroups was compared between the DCM and the mixed layer (Fig. 6B). The result indicates that this increase is only significant for chloroplast-related PUFAs.

To further assess the impact of this increase in chloroplast-related PUFAs on the vertical distribution of essential fatty acids, such as eicosapentaenoic acid (EPA), the depth at which EPA reaches its maximum concentration was compared to the DCM depth (fig. S12). The result shows that EPA frequently peaks near the DCM in tropical and subtropical oceans.

## DISCUSSION

### Structurally diverse eigenlipid clusters as an indicator of environmental drivers and microbial sources

The application of WGCNA to the global ocean lipidome has led to the identification of structurally distinctive EL clusters that are correlated with environmental factors. Given that there is no a priori reason for WGCNA to differentiate between structurally different lipids, the observed structural heterogeneity among ELs suggests that the lipid composition reflects the environmental conditions within the water column. It remains unclear whether such link arises from microbial adaptation to dynamic environmental conditions or from the presence of distinct microbial communities inhabiting the water column. However, the strong positive correlation of EL16 with divinyl chlorophyll-*a*—a pigment predominantly associated with the cyanobacterium *Prochlorococcus*—and with the total biovolume of *Prochlorococcus* in the water column potentially suggests a substantial contribution from *Prochlorococcus*. In contrast, EL8 appears to be a generalized signal from diverse phytoplankton taxa, as suggested by its positive correlation with chlorophyll-*a* (fig. S8A), a pigment used by a broad range of phytoplankton. In addition, the predominance of TAGs with PUFAs in EL1 and EL6 could potentially suggest a major contribution from eukaryotic phytoplankton, which have been reported as the primary producer of TAGs in the ocean (*25*, *26*). Therefore, it appears that the ELs may serve not only as markers of microbial adaptations to environmental changes but also, to some extent, as indicators of distinct microbial sources in various marine environments.

### Cold environments favor shorter acyl moieties

Homeoviscous adaptation by the desaturation of fatty acid chains in glycerolipids in response to decreasing temperature has been well documented (*10*) and observed on a global scale (*11*). In addition, chain shortening has been observed in laboratory cultures. For example, chain shortening has been observed for polar lipids in certain marine *Synechococcus* strains, which can shorten highly saturated acyl chains. Breton *et al.* (*27*) and Pittera *et al.* (*28*) reported that these cyanobacteria increase the C_14_/C_16_ fatty acid ratio in glycolipids under low growth temperatures. This observation aligns with the carbon number shift observed in the SFA-containing lipids from tropical to polar oceans, where the average chain length decreases from 15 to 14.2 carbon atoms per fatty acid (Fig. 4A). A similar trend is also observed for PUFA-containing lipids, with shorter chain lengths prevalent in colder environments (Fig. 4B). This suggests that, in addition to an increase in unsaturation highlighted in previous studies (*10*, *11*), colder environments favor shorter acyl moieties, and these effects are evident on a global scale. This preference for shorter-chain fatty acids can be attributed to their lower melting transition temperature compared to longer-chain fatty acids (*29*, *30*), allowing them to remain in a fluid state at lower temperatures.

Nonetheless, it remains uncertain whether such difference in chain length indicates that marine plankton are capable of shortening fatty acyl chains as part of their homeoviscous adaptation, or if plankton that produce shorter fatty acids are inherently better adapted to cold environments since studies have shown that psychrophiles tend to have shorter acyl chains than mesophiles (*31*, *32*). However, regardless of the mechanism responsible for regulating the chain length of lipids, it is not limited to the extremely cold polar ocean but is also evident in tropical to subtropical oceans (Fig. 4). Hence, it can be inferred that alterations in lipid chain length may serve as a universal adaptation strategy, alongside lipid unsaturation, for marine plankton as a collective response to temperature changes.

### Accumulation of non-phosphorus lipids in warm waters

Tropical and subtropical open oceans generally exhibit stratification, resulting in limited nutrient availability at the surface (*33*). A pertinent example is the Sargasso Sea, characterized by extremely depleted phosphate, measuring at less than 10 nM (*34*). In response to such phosphate scarcity, plankton have been observed replacing phospholipids with various non-phosphorus lipids to reduce their phosphorus requirements (*35*, *36*). None of the previously reported substitute lipids have been detected in the lipid cluster unique to the Sargasso Sea, i.e., EL11. Nonetheless, EL7, which predominantly contain non-phosphorus lipids and exhibits a stratified vertical distribution pattern at sea surface, show relatively high abundance in this region (fig. S2G). Given that EL7 contains lipids reported as substitute lipids used by phytoplankton in the Sargasso Sea (*35*), it is expected that its increased intensity in this region is attributable, in part (fig. S10D), to the substitution of phosphorus-containing lipids with their non-phosphorus counterparts, such as betaine lipids (fig. S11A).

Notably, the accumulation of glycolipids MGDG and SQDG containing SFA/MUFA chains at the sea surface is predominantly driven by warm temperature rather than limited phosphorus availability (Fig. 5). These glycolipids are the dominant lipid species in cyanobacteria (*37*), and their enrichment in warm oceans likely reflects the increasing dominance of cyanobacterial in these environments (*38*, *39*). Alternatively, studies have shown that these lipids can help preserve the functionality of thylakoid membranes of phytoplankton upon exposure to heat and light (*40*, *41*). This may explain the stratified vertical distribution pattern of EL7 at the sea surface, where light intensity is stronger than at the subsurface. Consequently, we propose that the accumulation of these glycolipids at warmer temperatures may result from the ecological dominance of cyanobacteria over eukaryotic phytoplankton and/or from the physiological response of phytoplankton to maintain photosynthetic capacity. Furthermore, these non-phosphorus lipids are generally less efficiently exported to the deeper ocean compared to phospholipids (*42*, *43*). It is plausible that this accumulation of glycolipids in warm temperature could influence the elemental stoichiometry of the biological pump in a progressively warmer future ocean.

### DCM: A crucial PUFA reservoir in tropical and subtropical oceans

The higher intensity of chloroplast lipids, which are the dominant lipids in EL8 and EL16, in DCM compared to sea surface could either be attributed to increasing phytoplankton biomass or the photoacclimation of phytoplankton by the increasing of cellular chlorophyll content (*44*–*46*). Notably, while both EL8 and EL16 show an increase in the DCM zone of tropical and subtropical oceans, their responses differ. The PUFA-containing, chlorophyll-related EL8 cluster shows a steeper increase from the surface to the DCM compared to the SFA/MUFA-containing, divinyl chlorophyll-related EL16 cluster (Fig. 6A). This more pronounced increase in EL8 may be linked to its higher PUFA content as PUFAs have been shown to increase under low-light conditions (*47*–*49*). This is supported by the significant increase of chloroplast lipids–related PUFAs in DCM, while such increase is not observed for other lipid classes (Fig. 6B). Furthermore, the difference in response between EL8 and EL16 could reflect their distinct associations with specific chlorophyll types. It is possible that the photosystems associated with divinyl chlorophyll may differ in their adaptation to low-light conditions compared to those associated with chlorophyll as the former is inherently more efficient at harvesting blue light at deeper parts of the water column (*50*).

Nevertheless, this increased PUFA content in the DCM zone suggests that this zone could be an important PUFA (e.g., essential fatty acids) reservoir in the tropical and subtropical ocean food webs. Future ocean warming is expected to reduce vertical mixing (*51*), leading to increased variability in DCM. This changing ocean stratification may influence PUFA production and availability in tropical and subtropical marine ecosystems. These findings underscore the necessity for ongoing research to elucidate the mechanisms governing PUFA production and fate. Such knowledge will be critical for predicting and managing future shifts in marine lipid distributions and their ecological impacts.

## MATERIALS AND METHODS

### Samples and instrumental analysis

In this study, we used the mass spectrometry data from Holm and Van Mooy (*20*) where the procedures for sample collection, extraction, and instrumental analysis have been described in detail. In brief, suspended particulate matter was collected at 146 sampling locations (indicated as dots in Fig. 3A) on seven research cruises. A total of 930 lipids extracts were extracted from the planktonic particles collected on 0.22-μm pore-size membranes using a modified Bligh and Dyer method as described by Popendorf *et al.* (*52*). Spectra were generated by the analysis of lipids extracts using high-performance liquid chromatography coupled with electrospray ionization high-resolution accurate-mass mass spectrometry.

### Data treatment

The code used for data processing, as well as the intermediate and final datasets generated, is publicly accessible through Zenodo (*53*). The data processing workflow is outlined below.

#### Feature extraction

In accordance with the data processing steps outlined in Holm *et al.* (*11*), we used XCMS (*54*) with identical parameters as those specified in the publication. R package XCMS was used for feature extraction from a total ion chromatogram, including peak grouping, retention time correction, and peak alignment. In addition, MS^2^ spectra were collected for 1339 lipid species using XCMS with the maxTIC option.

#### Blank subtraction

To remove potential contaminant signals, the peak intensities of detected features in samples were subtracted from those in blanks. Features that exhibited median peak intensities less than 20-fold higher in the samples compared to the blanks were excluded from the analysis.

#### Feature grouping and annotation

R package CAMERA (*55*) was used in this study to first annotate potential adducts, isotopologs, and in-source fragments. Lipid species were then annotated and categorized as follows:

Ann.1. Annotation provided in Holm *et al.* (*11*) that includes various classes of ester-linked glycerolipids as listed in table S1.

Ann.2. Annotations by a modified version of LOBSTAHS (*21*), which extends the library to include ether-linked glycerolipids and lipids curated in LIPIDMAPS Structure Database (*22*).

Ann.3. Annotations by matching MS^2^ spectra of lipid species against MS-DIAL (*56*) database and GNPS (*16*) database with Python package matchms (*57*).

Ann.2 and Ann.3 lipids included ether-linked glycerolipids, photosynthetic pigments (e.g., chlorophylls and carotenoids), sphingolipids, ornithine lipids, etc. Their annotation accuracy is discussed in text S2. The detailed lipid annotations are available on Zenodo (*53*).

#### Weighted correlation network analysis

The peak intensity of each lipid species was normalized to the total intensity of all annotated lipids and then transformed using a centered log-ratio approach. Subsequently, a WGCNA analysis was conducted separately for samples from each ocean region (i.e., the Atlantic Ocean, the Antarctic, and the Pacific Ocean; text S3). Detailed information about this analysis is provided in text S4. ELs were derived from samples collected in the Atlantic Ocean, with samples from the Antarctic and the North Pacific incorporated after the WGCNA analysis. A permutation test was conducted to quantify the reproducibility of these ELs using an in-group proportion score (*58*). In addition, a separate analysis applying WGCNA to samples collected from the mixed layer was performed to validate WGCNA’s usefulness for uncovering the collective planktonic response to varying environmental conditions (text S5).

### Meta-clustering of EL clusters

Two additional clustering analyses were performed following the WGCNA analysis: one based on the geographical profiles of ELs within the framework of Longhurst biogeochemical provinces (*23*) and another based on their depth profiles. A meta-clustering was then conducted to integrate the results of all three clustering approaches.

### Statistical analysis

The cluster membership score of lipid species in each cluster was determined on the basis of Spearman’s rank correlation to the respective cluster. Pairwise statistical significance for depth profiles and prevalent Longhurst provinces was assessed using the Mann-Whitney *U* test, using a two-sided test for depth profiles and a one-sided test for Longhurst provinces. Effect sizes were standardized using Cliff’s Delta. Significant enrichment in the number of lipid classes within ELs was evaluated using a Chi-square goodness-of-fit test, which compared observed counts of lipid species categorized by headgroup within each EL to an expected uniform distribution across all ELs. Statistical significance for differences in lipid distributions between the mixed layer and the DCM was evaluated using a two-sided *t* test. Effect sizes were standardized using Cohen’s *d*. For all tests above, *P* values were adjusted for multiple comparisons to control the false discovery rate. The significance of meta-clustering was assessed using Monte Carlo simulations with the R package sigclust2. Cophenetic correlation was used to measure the consistency of hierarchical clustering by comparing the original pairwise dissimilarities with those implied by the clustering dendrogram. Structural dissimilarity among EL clusters was evaluated using mixed-effects modeling, treating structural dissimilarity as a fixed effect and sample-specific variation as a random effect. The significance of model coefficients was assessed using *z* statistics. PCA was used to evaluate the relationships between available environmental parameters and the relative intensity of ELs. Random Forest regression models were used to assess how specific environmental parameters (e.g., PO_4_ concentration and in situ temperature) relate to lipid intensities. Models were trained with a 70:30 train-test split, and performance was assessed using *R*^2^ scores. Feature importance, scaled by *R*^2^, quantified the contribution of each predictor. All statistical analyses were performed in Python or R.
