# Supplementary material for: Unraveling plankton adaptation in global oceans through the untargeted analysis of lipidomes
Source: Sci Adv. 2025 May 23;11(21):eads4605. doi: 10.1126/sciadv.ads4605 (PMC12101493; doi:10.1126/sciadv.ads4605)
Supplement: Supplementary file 1 — Supplementary Text S1 to S5 Figs. S1 to S16 Tables S1 to S3 [file sciadv.ads4605_sm.pdf]

Science Advances
AAAS

Supplementary Materials for

Unraveling plankton adaptation in global oceans through the untargeted analysis of lipidomes

Weimin Liu *et al.*

Corresponding author: Weimin Liu, wliu@marum.de

Sci. Adv. **11**, eads4605 (2025)
DOI: 10.1126/sciadv.ads4605

This PDF file includes:

Supplementary Text S1 to S5
Figs. S1 to S16
Tables S1 to S3

Text S1: Eigenlipid clusters stability

Eigenlipid cluster stability was assessed through a permutation-based test, using the In-Group Proportion (IGP) to measure the reproducibility of cluster assignments. Specifically, 100 random perturbations were generated by bootstrap resampling the dataset, with rows (samples) selected with replacement to maintain the original dataset dimensions. For each perturbed dataset, eigenlipid clusters were reconstructed using the same parameters as in the original analysis. The IGP for each cluster was calculated as the proportion of lipid species in the original cluster that remained assigned to the same cluster (or its best-matching counterpart) across perturbations. Higher IGP values indicate greater stability of the eigenlipid clusters.

To assess statistical significance, a null distribution of IGP values was generated by randomly shuffling cluster assignments and recalculating IGP. p-values were computed as the proportion of null IGP values exceeding or equaling the observed IGP for each module. Observed IGP values consistently exceeded the null distributions, resulting in p-values < 0.001 for all clusters. This indicates that the observed clustering is highly significant.

The resulting IGP values for 16 eigenlipid clusters (EL1 to EL16) and the non-cluster (EL0) are shown in Fig. S13. Clusters EL3, EL4, EL7, EL9, EL10, EL12, and EL16 showed strong preservation, with more than 80% of their lipid species consistently clustered together across perturbations. The remaining clusters demonstrated moderate stability, with more than 50% of their lipid species remaining consistently assigned.

Throughout the results and discussion, only lipid species with a cluster membership score (kME, measured as the Spearman correlation between the intensity profile of a lipid species and the corresponding eigenlipid cluster) greater than 0.5 were retained for interpretation. The cluster membership scores for each lipid species are available in the Zenodo repository (53).

Text S2: Lipid annotations

An extended library was used to annotate the Ann.2 lipids. Only case codes C2a and C2b (multiple adducts observed) reported by LOBSTAHS were included in the final annotation. However, it should be noted that the adduct hierarchy for lipids, which LOBSTAHS relies upon, cannot be comprehensively validated within the extended library. As a result, annotations generated using this extended library are considered accurate only at the chemical formula level.

Due to varying confidence levels associated with different annotation methods, the discussion of specific lipid clusters primarily focuses on Ann.1 lipids. The elemental compositions discussed are derived from a combination of Ann.1 and Ann.2 lipids. Ann.3 lipids are included to confirm molecular structures when necessary—such as in the case of chlorophylls—and to establish connections between the co-expression network and the molecular network generated using GNPS.

It is essential to emphasize that the primary objective of this study is not to achieve precise lipid annotations. This level of precision is not required for the co-expression network analysis. Instead, the feature annotation step serves as a reference framework.

Text S3: Batch effects

The samples analyzed in Holm et al. (11) were collected from various cruises and regions, including the Atlantic Ocean, the Antarctic Ocean, and the Pacific Ocean. Given the substantial time span over which these samples were measured, the possibility of batch effects cannot be ruled out. Consequently, it is difficult to determine whether the variances observed among geological batches genuinely reflect biogeochemical signals or are instead attributable to batch effects. To minimize potential interference from batch effects, weighted correlation network analysis was conducted separately for each geological batch. Our primary focus was on the Atlantic batch, which contains the largest sample size and the most extensive range of biogeochemical variances. Samples collected from the Antarctic and the North Pacific were incorporated after the WGCNA analysis.

Text S4: WGCNA analysis

The R package WGCNA (version 1.69) was employed. Hierarchical clustering was first performed using the *hclust* function from the *stats* package to assess the presence of outliers. After removing the outlier samples, a range of soft-thresholding powers (1–20) was evaluated to identify the optimal value, utilizing the *pickSoftThreshold* function from the WGCNA package. As a result, a soft-thresholding power of 12 was chosen. The network was constructed using the *blockwiseModules* function from the WGCNA package, utilizing a signed network type and the *bicor* correlation function. Additional parameters included a maximum p-value for outlier identification of 0.05 and a minimum module size of 20. Module information, including the assigned colors and module eigenvalues, was saved as CSV files in the Zenodo data repository (53).

Text S5: Validating WGCNA: Unravelling the link between lipid unsaturation and temperature in surface mixed layer.

The principal component analysis (PCA) biplot shown in Fig. S14 provides a comprehensive visualization of the interplay between eigenlipid distribution and environmental factors in the mixed layer. Notably, PC1, which accounts for 44.26% of the total variance of the lipidome in the mixed layer, emerges as a pivotal determinant. Further investigation reveals that temperature is the predominant factor contributing to PC1, establishing a clear relationship between eigenlipid composition and temperature. This underscores the potential impact of this environmental factor on lipidome dynamics. Among all eigenlipids, EL0 exhibits a near-orthogonal relationship with PC1, indicating its relative stability amidst temperature changes. In contrast, EL1 shows a positive correlation with PC1, suggesting its enhanced prevalence in colder oceans. Conversely, EL2 displays a negative correlation with PC1, indicating its higher abundance in warmer oceans (Fig. S15).

Fig. S16 illustrates the partitioning of lipid species annotated by Holm et al. (11) within lipid clusters in this study. Notably, except for GADG, there is a decrease in the number of double bonds from eigenlipids negatively correlated with temperature to those positively correlated with temperature. This pattern aligns with the findings of Holm et al., which suggest that glycerolipids

relatively enriched under colder temperatures tend to be more unsaturated. The agreement between our findings and those of Holm et al. validates that WGCNA can discern and elucidate the impact of environmental factors on lipidome co-expression.

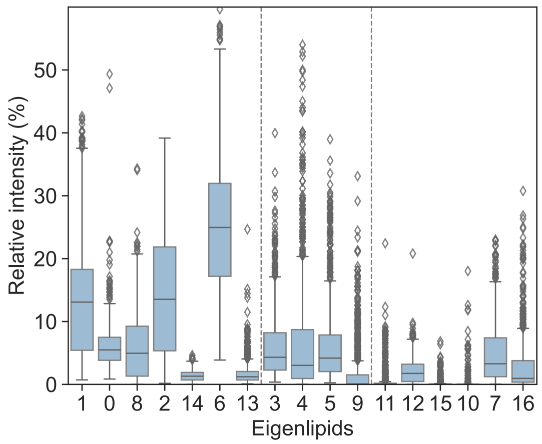

Fig. S1. Relative intensity of eigenlipid clusters across samples. The relative intensity is calculated as the ratio of a given eigenlipid cluster's intensity to the total intensity of all annotated lipid species in that sample. In each boxplot, the central line represents the median, the box spans the interquartile range (IQR; 25th to 75th percentiles), and the whiskers extend to the most extreme data points within 1.5× the IQR. Points outside this range are plotted individually as outliers. The vertical lines divide the three metaclusters.

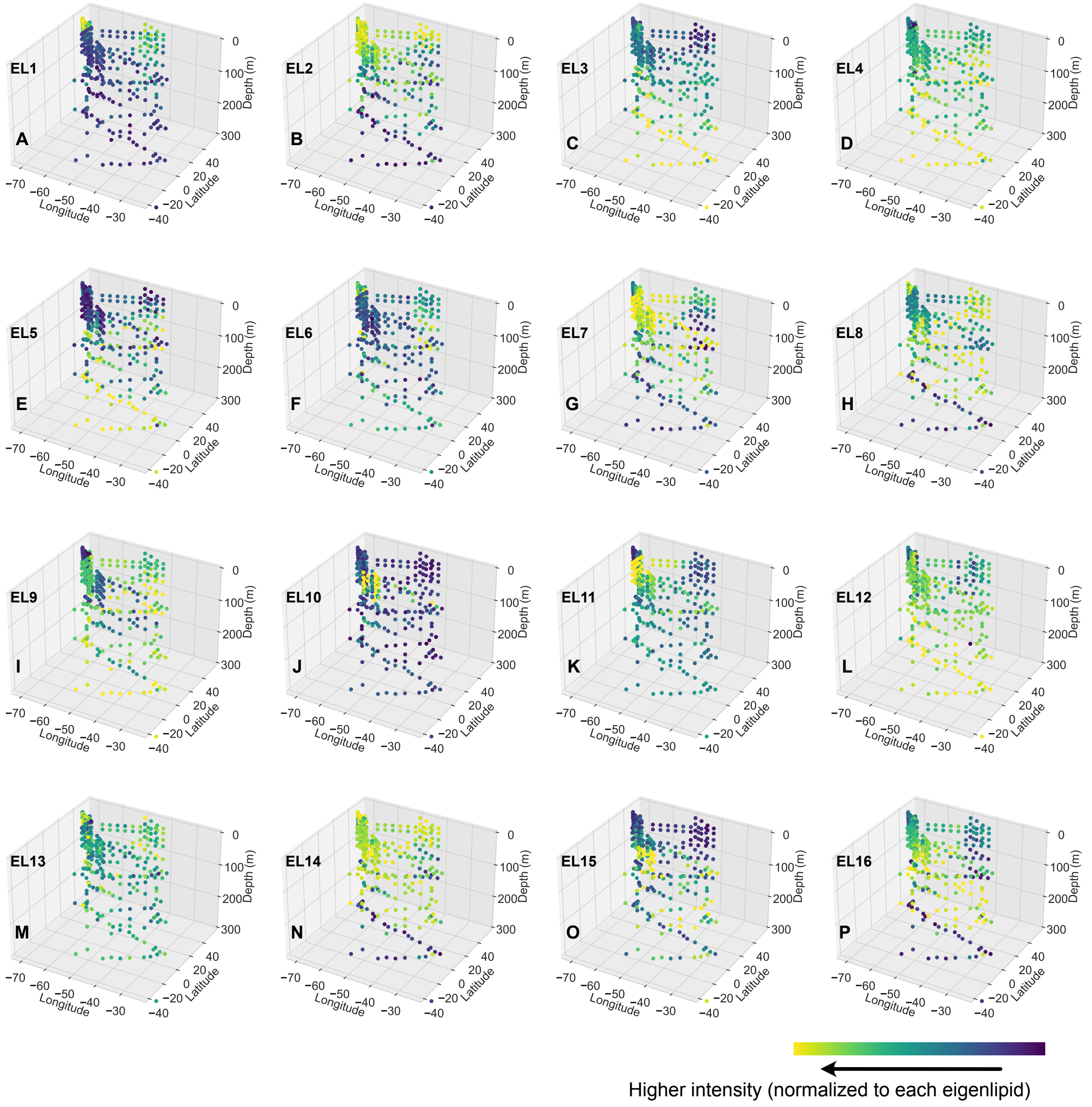

Fig. S2. Spatial distribution of eigenlipids identified throughout the sampled water column in the Atlantic. The color scale indicates the eigenvalue assigned to each eigenlipid cluster.

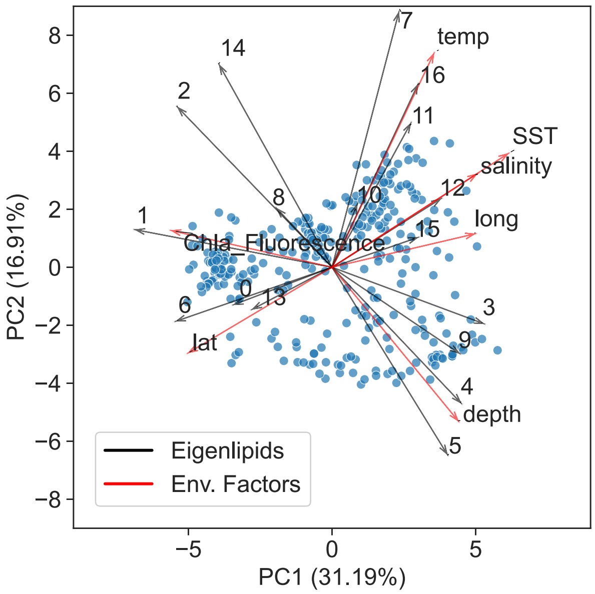

Fig. S3. Principal Component Analysis (PCA) biplot of eigenlipids identified throughout the sampled water column. Only samples with complete environmental data (shown as red vectors) were included (n = 319). The biplot displays the first two principal components (PC1 and PC2), which accounts for 31.19% and 16.91% of the total variance, respectively. Each point represents a sample, while the direction and length of the vectors indicate each variable's contribution to overall variability. Black vectors represent the eigenlipids. Note: temp = in-situ temperature, SST = sea surface temperature, lat = absolute value of latitude, long = longitude.

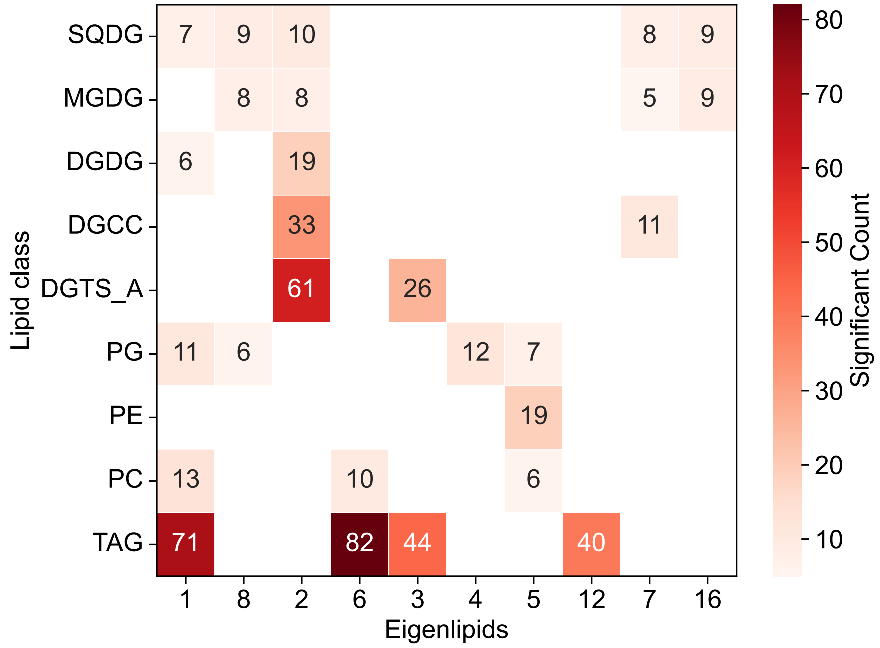

Fig. S4. Significant enrichment in the number of Ann.1 lipid species categorized by headgroups across eigenlipids. Observed counts are displayed for eigenlipids where lipid classes are significantly overrepresented. Rows represent lipid classes, and columns represent eigenlipids. The heatmap is color-coded by significant counts (adjusted $p < 0.05$), with blank cells indicating non-significant results. Statistical significance was determined using a Chi-square goodness-of-fit test against an expected uniform distribution.

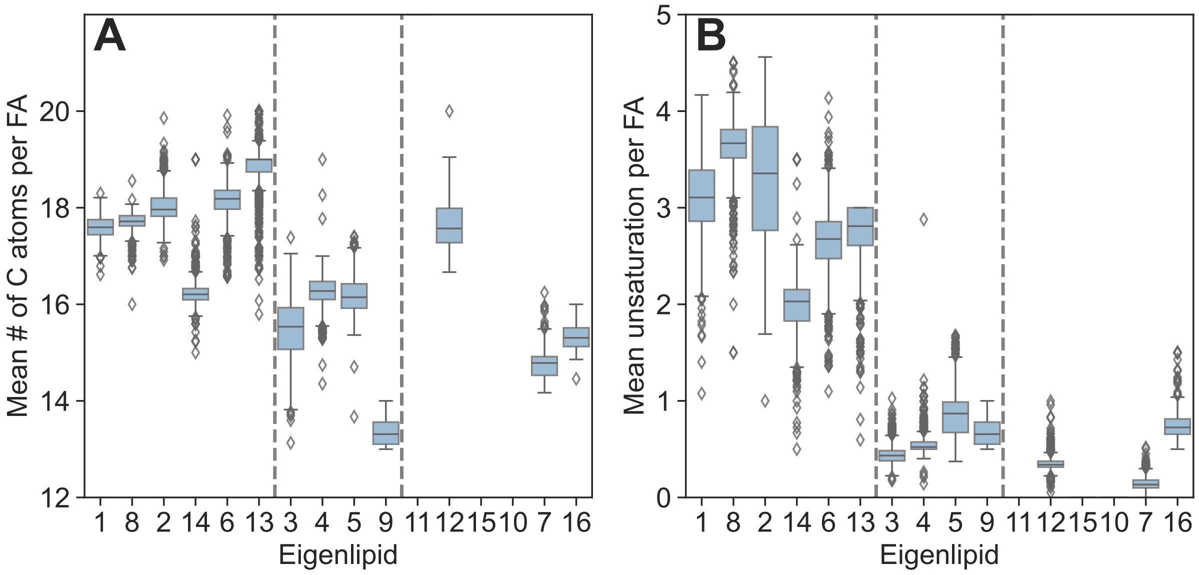

Fig. S5. Distribution of the weighted mean carbon number (A) and unsaturation (B) per fatty acid chains of glycerolipids in each eigenlipid identified throughout the sampled water column. The x-axis represents eigenlipid clusters identified by WGCNA. The vertical lines divide the three metaclusters.

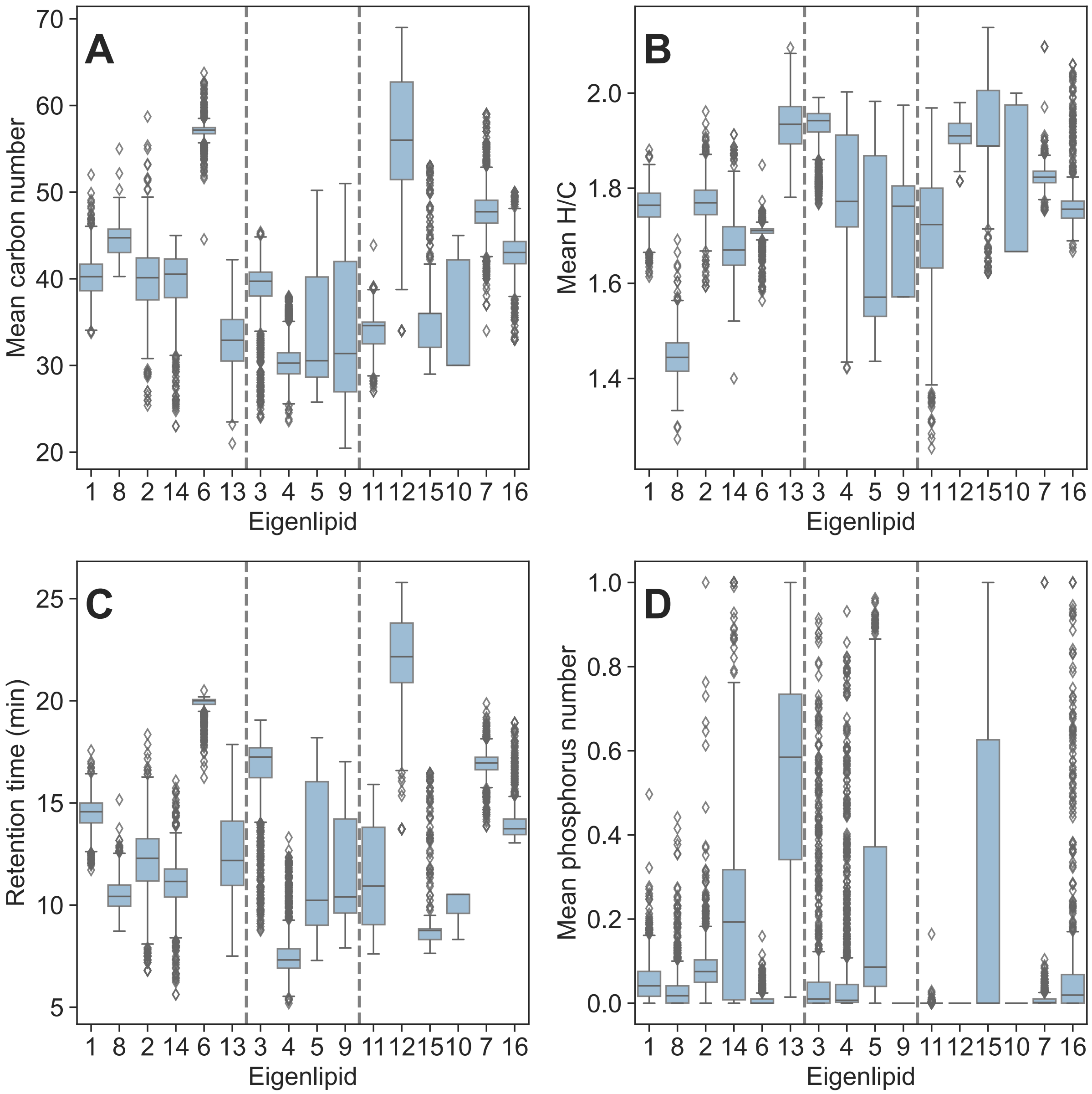

Fig. S6. Molecular properties of Ann.2 lipids in each eigenlipid cluster identified throughout the sampled water column, including the weighted mean carbon number (A), weighted mean hydrogen-to-carbon ratio (B), weighted mean retention time (C), and weighted mean phosphorus number in the formula (D). The vertical lines divide the three meta-clusters.

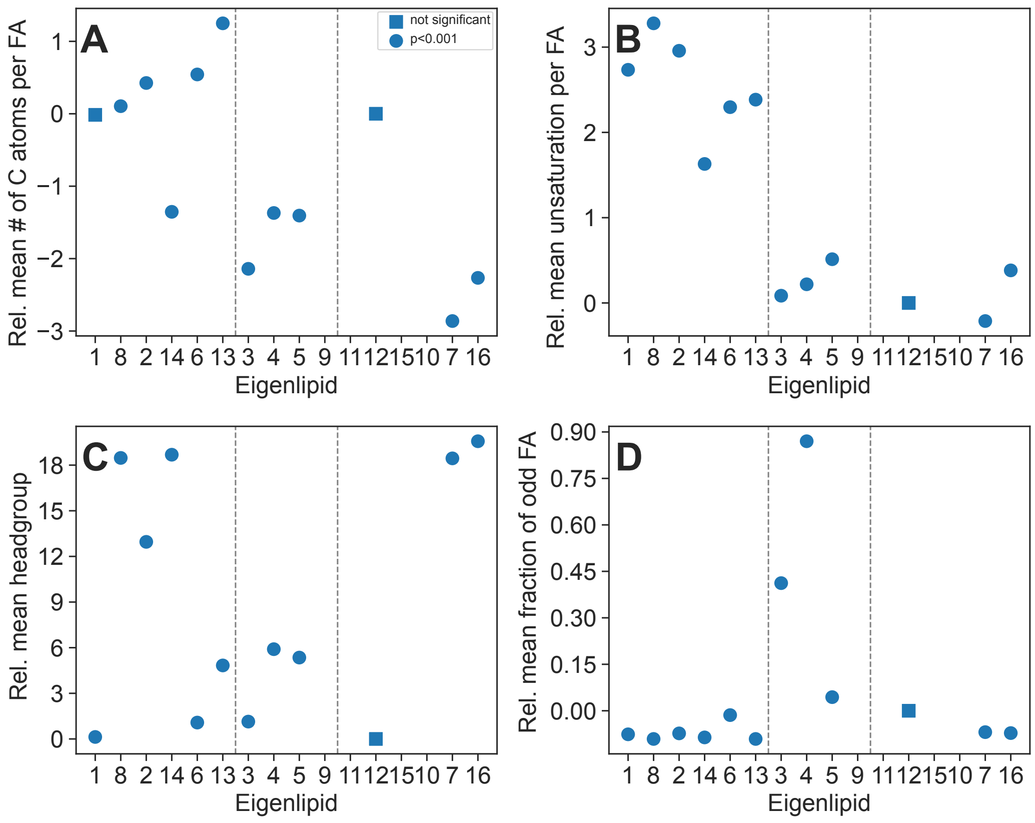

Fig. S7. Structural dissimilarity among eigenlipid clusters. This figure compares structural characteristics of eigenlipid clusters relative to EL12, which is dominated by saturated/monounsaturated triacylglycerols. The vertical lines divide the three metaclusters. Relative differences were calculated using mixed-effect modeling, with fixed effects modeling differences between eigenlipid clusters and random effects accounting for sample variation. Panels show the weighted mean: (A) number of carbon atoms per fatty acid (FA), (B) unsaturation per FA, (C) headgroup type, and (D) fraction of odd-numbered FAs across all samples. Significant results are represented by dots, while non-significant results are represented by squares. Lipid headgroups are encoded as follows: TAG = 0, PC = 4, PE = 5, PG = 6, DGTS/A = 10, DGCC = 11, DGDG = 18, MGDG = 19, SQDG = 20. The fraction of odd-numbered FAs assumes the presence of odd FA only when the total carbon number is odd, likely underestimating their true proportion.

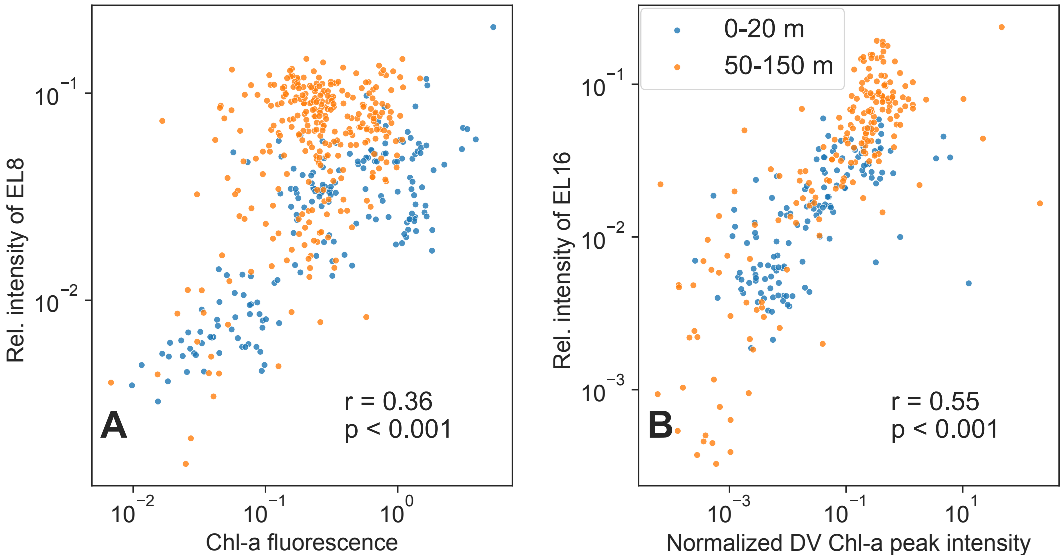

Fig. S8. Relationship between chlorophylls and the DCM-related eigenlipids EL8 and EL16. (A) EL8 vs. Chl-*a* fluorescence and (B) EL16 vs. normalized divinyl Chl-*a* peak intensity. Spearman's rank correlation coefficient (r) and corresponding p-value indicate a significant monotonic relationship in both cases. Divinyl peak intensity is determined from the extracted ion chromatogram peak at *m/z* 891.5269, with a characteristic fragment ion at *m/z* 553.21. Peak intensity is normalized by the peak intensity of internal standard. Data points from cruise RR1813 and LMG1810 are excluded as the presence of divinyl Chl-*a* cannot be confirmed by MS^2 spectra.

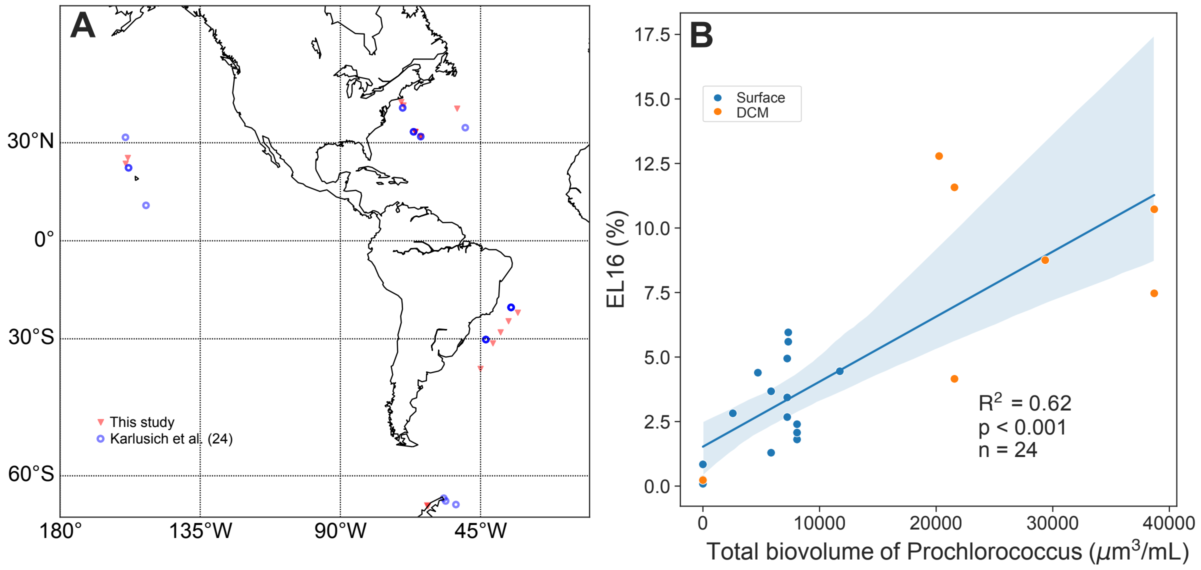

Fig. S9. Comparison of the relative intensities of EL16 with the total biovolume of *Prochlorococcus* from Karlusich et al. (24). (A) Map showing sampling locations from this study and those from Karlusich et al. (24) used for comparison. (B) Relationship between the relative intensity of EL16 and the total biovolume of *Prochlorococcus*. Biovolume is calculated as the product of average cell size and cell count. The blue line represents the linear fit. Insets show the correlation coefficient (R^2) from the linear fit and the p-value from the F-test (n = 24). Samples collected from the DCM and the surface are shown in different colors.

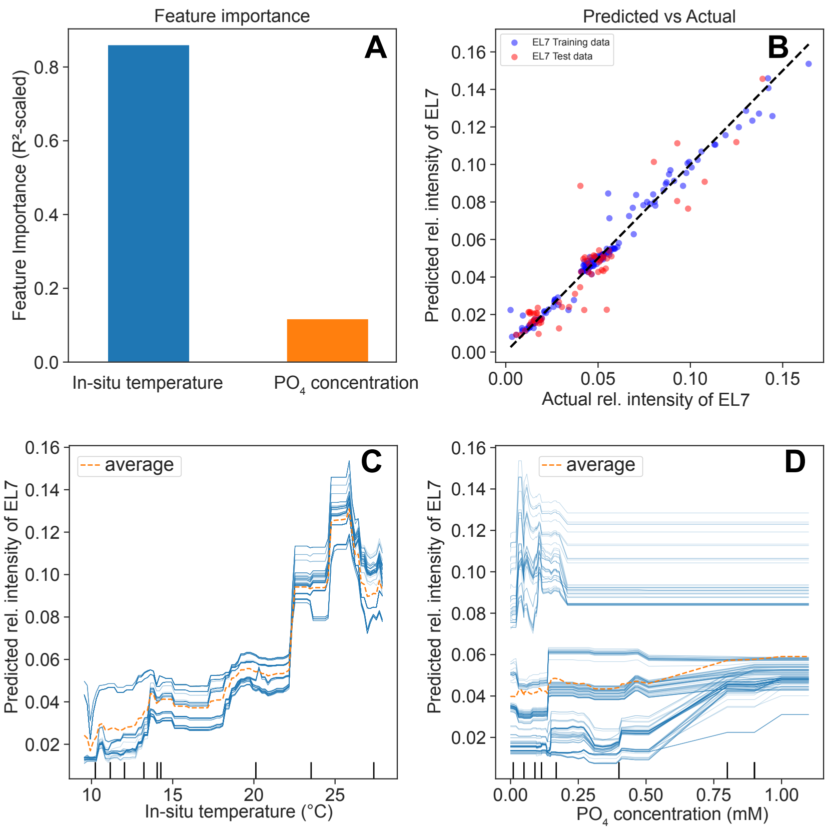

Fig. S10. In-situ temperature as a strong predictor for the relative intensity of EL7, a phosphorus-depleted eigenlipid cluster prevalent in the surface of tropical and subtropical oceans. (A) Feature importance of PO_4 and in-situ temperature for predicting EL7, scaled by the Random Forest model's R^2 value. (B) Predicted vs. actual relative intensities of EL7 based on the Random Forest model. (C) Individual Conditional Expectation (ICE) plot showing the predicted relative intensity of EL7 as a function of in-situ temperature, with PO_4 concentration held constant. Each curve represents the trajectory of an individual sample. (D) ICE plot showing the predicted relative intensity of EL7 as a function of PO_4 concentration, with in-situ temperature held constant.

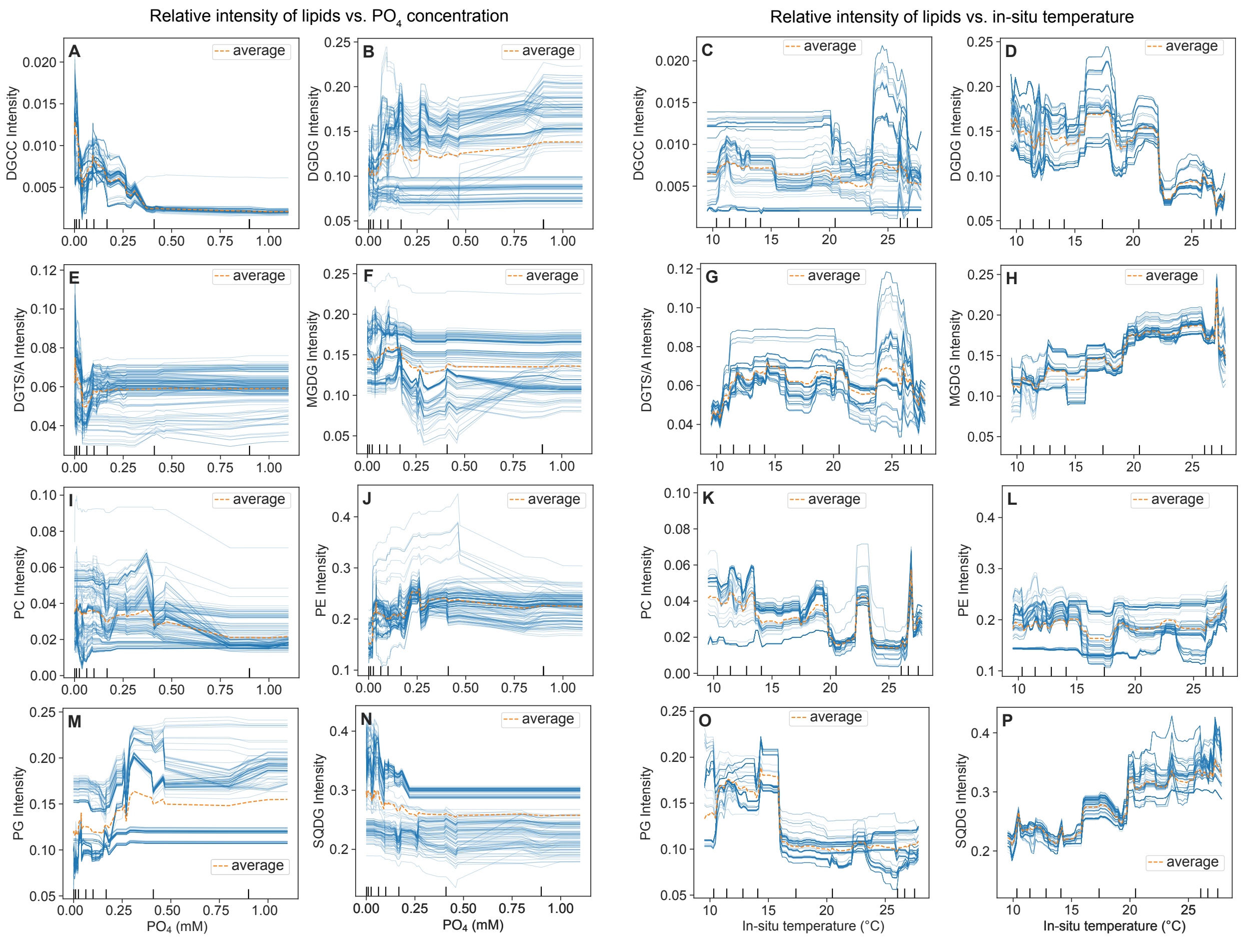

Fig. S11. Individual Conditional Expectation (ICE) plots illustrating the relationship between the relative intensities of lipids and PO_4 concentration (A, B, E, F, I, J, M, and N) or in-situ temperature (C, D, G, H, K, L, O, and P) at the sea surface, as predicted by Random Forest regression models. The relative intensity of a given lipid classes is calculated by normalizing the intensity of the lipid class with SFA/MUFA chains to the total intensity of all lipids with SFA/MUFA chains. ICE plots show how the predicted lipid intensity changes as a single feature (PO_4 concentration or in-situ temperature) varies, while the other feature is held constant. Each curve represents the trajectory of an individual sample.

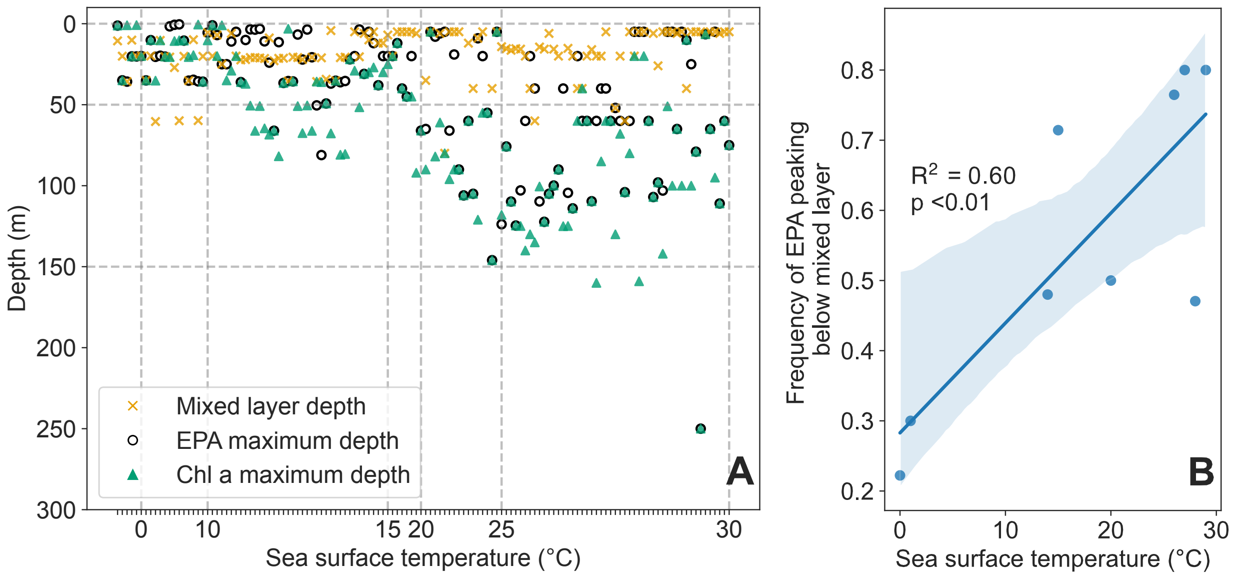

Fig. S12. EPA peaks more frequently below the mixed layer near chlorophyll-*a* maximum in warmer temperature. (A) Comparison of mixed layer depth and EPA maximum depth across global oceans. The x-axis represents individual sampled water columns, sorted by sea surface temperature. Open circles indicate the depth at which the concentration of EPA-containing species peak in each water column, 'x' marks indicate the mixed layer depth, and triangles denote the depth at which chlorophyll-*a* concentration peaks. (B) Increasing frequency of EPA peak below the mixed layer with increasing sea surface temperature. Only sea surface temperature covered by more than three sampling sites is included. The blue line represents the linear fit, with the inset showing the coefficient of determination (R^2) for the linear regression fit using ordinary least squares and the p-value from the F-test (n = 9).

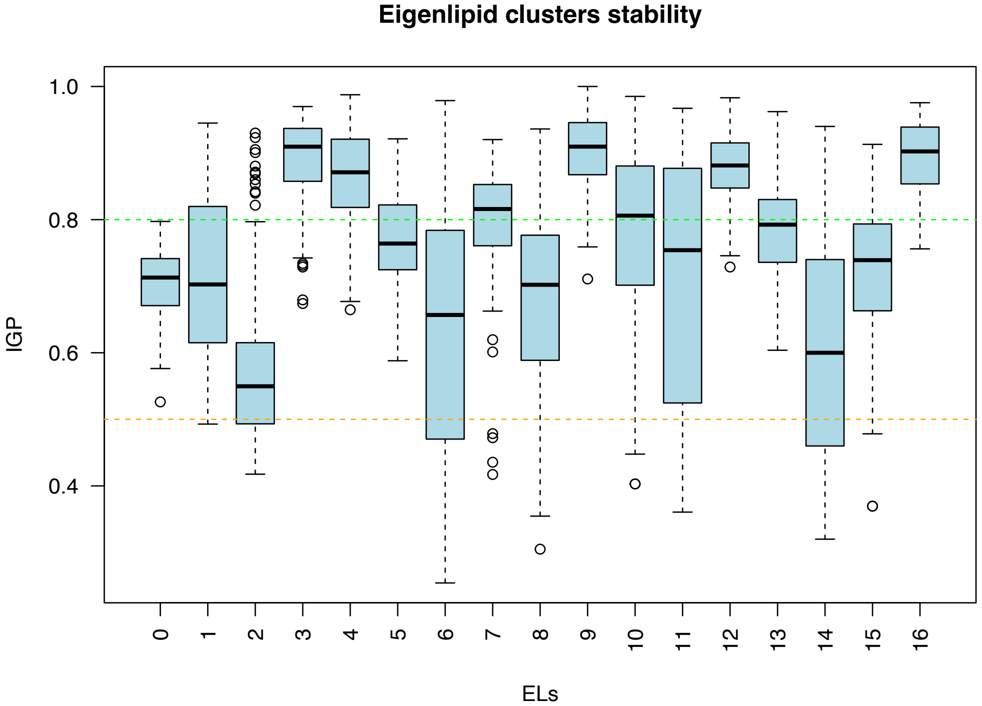

Fig. S13. Stability of eigenlipid clusters evaluated using permutation tests with bootstrapping. The stability metric, In-Group Proportion (IGP), was calculated for each cluster as the proportion of lipid species from the original WGCNA clustering results that remained consistently assigned to the same cluster (or its closest counterpart) across bootstrap perturbations. Higher IGP values indicate greater stability of eigenlipid clusters.

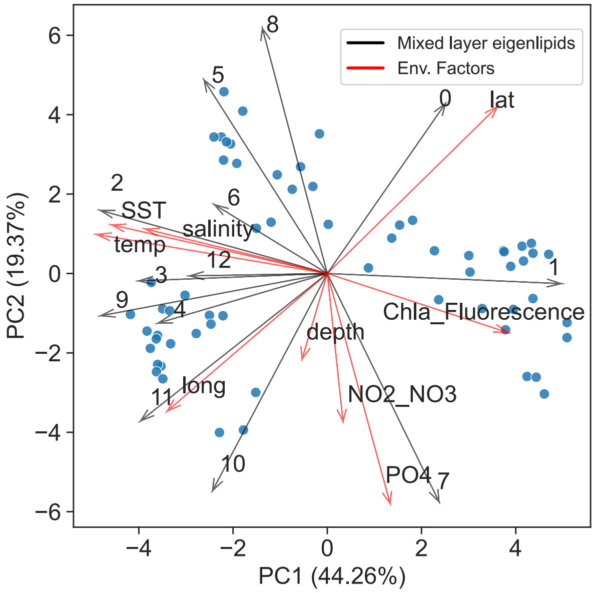

Fig. S14. Principal Component Analysis (PCA) biplot of mixed layer eigenlipids. The biplot displays the first two principal components (PC1 and PC2), which account for 44.26% and 19.37% of the total variance, respectively. Each point represents a sample taken from the Atlantic Ocean, and the direction and length of the vectors indicate the contribution and importance of the corresponding variables to overall variability. Black and red vectors represent eigenlipids and environmental factors, respectively. Note: temp = in-situ temperature, SST = sea surface temperature, lat = absolute value of latitude, long = longitude.

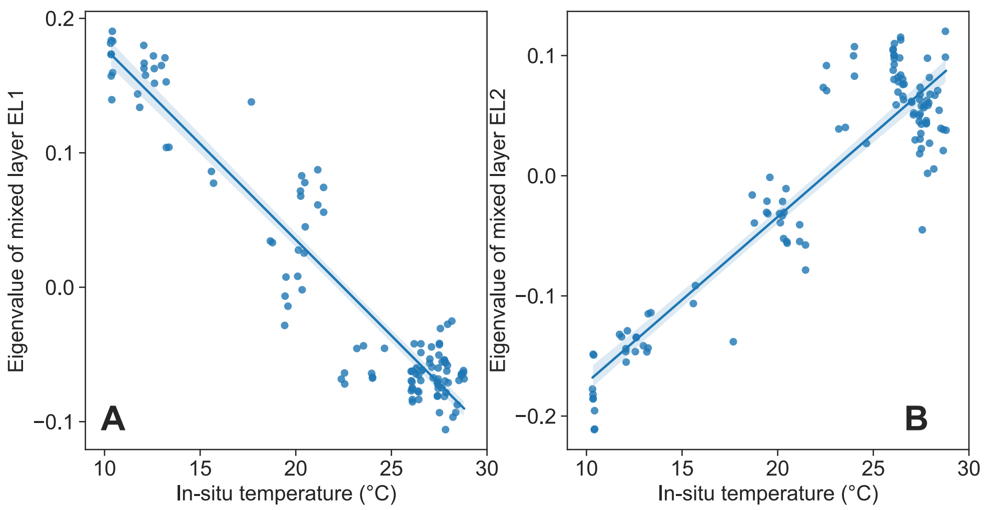

Fig. S15. Correlation between in-situ temperature and mixed layer eigenlipid 1 (A) and 2 (B). The y-axis represents the eigenvalue of the relative peak intensities of the corresponding eigenlipid cluster.

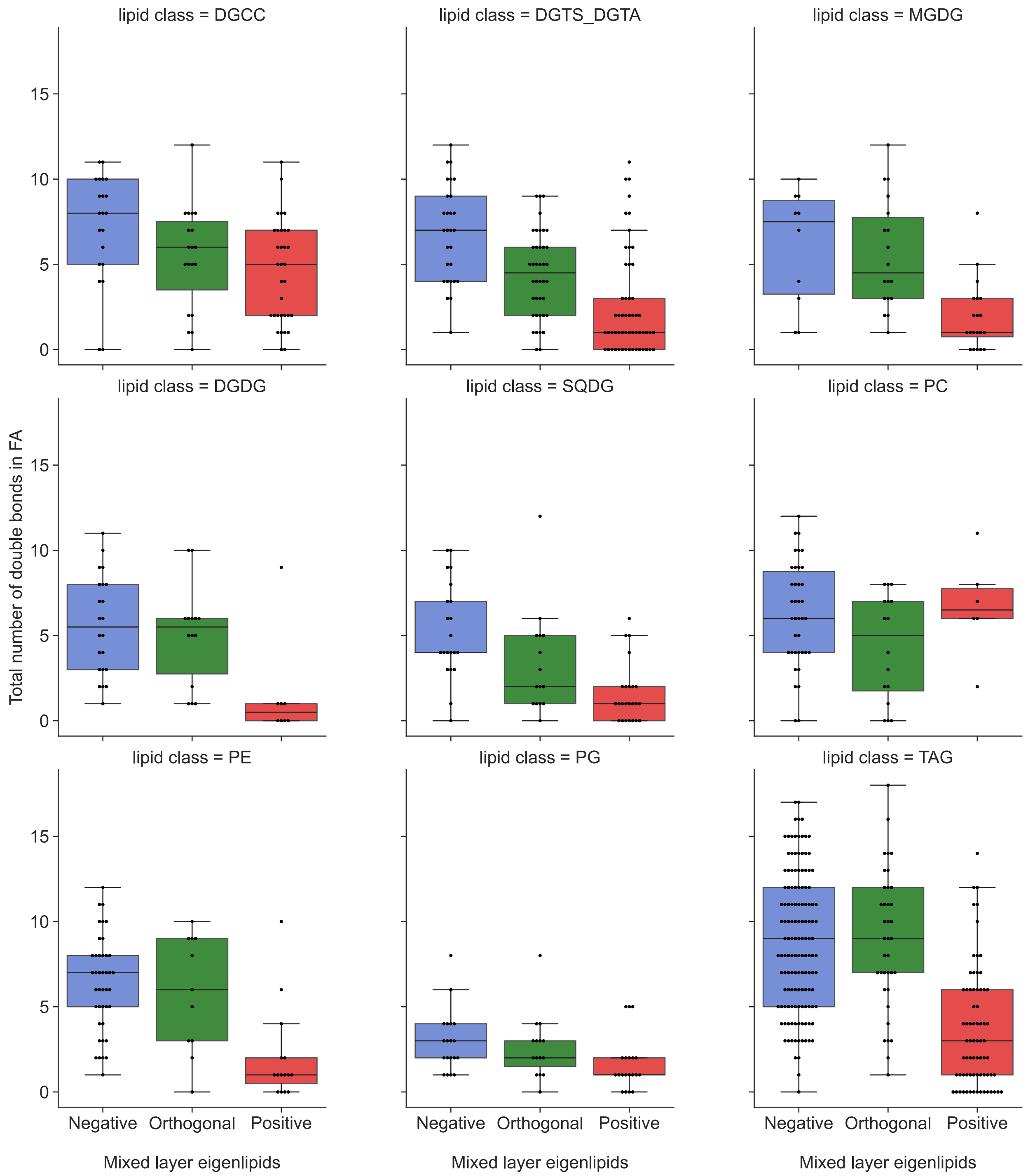

Fig. S16. Distribution of the total number of double bonds in the fatty acid chains of glycerolipids in each mixed layer eigenlipid. The x-axis represents the eigenlipid clusters identified through the WGCNA analysis: 'Negative' refers to eigenlipids negatively correlated with temperature, 'Orthogonal' refers to eigenlipids without significant correlation with temperature, and 'Positive' refers to eigenlipids positively correlated with temperature.

Table S1. List of abbreviations for Ann.1 lipid species analyzed.

Lipid category	Lipid class	Definition
Neutral lipids	TAG	Triacylglycerols
Betaine lipids	DGTS	Diacylglyceryl trimethylhomoserine
	DGTA	Diacylglyceryl hydroxymethyl-N,N,N-trimethyl-β-alanine
	DGCC	Diacylglyceryl-3-O-carboxyhydroxymethylcholine
Glycolipids	MGDG	Monogalactosyldiacylglycerol
	DGDG	Digalactosyldiacylglycerol
	SQDG	Sulfoquinovosyl diacylglycerol
Phospholipids	PC	Phosphatidylcholine
	PE	Phosphatidylethanolamine
	PG	Phosphatidylglycerol

Table S2. Dominant glycerolipid species across eigenlipid clusters. The table summarizes the total number of lipid species and the top five glycerolipids ranked by mean peak intensity and cluster membership score within each eigenlipid cluster. Lipid species with cluster membership score greater than 0.5 are included. If fewer than five lipid species met this criterion, the remaining entries are listed. If no lipid species met the criterion, it is denoted as '/'.

Metacluster	Eigenlipid cluster	Total lipid species	Top five glycerolipids by mean concentration and cluster membership
MC1	1	481	TAG 54:13, TAG 48:8, TAG 54:14. TAG 51:9. TAG 56:14
	8	120	MGDG 34:8, PG 34:6, SQDG 36:8, SQDG 36:9, PG 34:4
	2	424	MGDG 34:1, DGTS/A 36:6, DGCC 44:12, TAG 41:7, MGDG 34:2
	14	47	DGDG 34:6, DGDG 36:8, SQDG 32:3, MGDG 34:3, DGDG 34:7
	6	228	TAG 58:11, TAG 62:16, TAG 56:10, TAG 51:5, TAG 55:8
	13	52	PE 38:6, PE 40:6, PC 30:0, PC40:6
MC2	3	351	TAG 38:0, DGTS/A 36:1, TAG 36:0, TAG 47:0, TAG 38:1
	4	305	PG 38:2, PE 28:2, DGCC 36:6, PG 29:0, PE 31:1
	5	245	PG 38:0, PE 32:1, PE 30:2, PG 33:2, PG 37:0
	9	75	DGCC 28:2, DGTS/A 26:1
MC3	12	55	TAG 53:0, TAG 55:1, TAG 60:2, TAG 54:0, TAG 60:4
	15	46	/
	7	156	MGDG 32:0, MGDG 30:0, MGDG 28:0, DGTS/A 37:2, DGTS/A 28:0
	16	39	SQDG 30:2, MGDG 28:2, SQDG 29:1, MGDG 28:1, MGDG 30:2
	10	67	/
	11	56	DGTS/A 44:10

Table S3. Distribution of fatty acid composition in the form of total number of carbon atoms (25th percentile-75th percentile): total number of double bonds (25th percentile-75th percentile) grouped by glycerolipid classes in each eigenlipid cluster. Note that the values refer to the fatty acid moieties excluding the glycerol moieties.

ELs	DGCC	DGDG	DGTS/A	MGDG	PC	PE	PG	SQDG	TAG
1	36-38:8-10	38-40:3-9	31-37:4-8	/	34-38:8-10	32-36:8-10	39-41:2-3	31-37:3-6	48-54:6-13
2	36-40:5-9	34-36:3-8	33-40:3-8	34-34:1-9	32-33:4-5	/	/	32-33:0-5	/
3	/	/	37-43:0-1	/	39-40:3-5	33-34:1-1	/	/	40-47:0-2
4	/	/	/	/	/	28-31:1-2	31-35:0-2	/	/
5	/	/	/	/	/	28-32:0-2	33-37:0-2	/	/
6	/	/	/	/	37-44:6-10	37-41:4-10	31-32:1-2	/	51-60:5-11
7	34-37:1-2	29-30:0-0	30-39:0-4	30-32:0-1	/	/	/	29-31:0-0	/
8	/	/	/	34-36:6-9	/	32-36:5-7	34-34:4-4	34-36:5-9	/
9	29-30:0-2	/	/	/	/	/	/	/	/
10	/	/	/	/	/	/	/	/	/
11	/	/	/	/	/	/	/	/	/
12	/	/	/	/	/	/	/	/	53-58:1-3
13	/	/	/	/	32-38:2-4	38-40:6-6	/	/	/
14	35-37:5-6	32-34:3-7	/	34-36:3-4	/	/	/	32-33:3-4	/
15	/	/	/	/	/	/	/	/	/
16	/	/	/	28-30:2-3	/	/	/	29-31:1-2	/
